# Supplementary material for: Features of Protein Unfolding Transitions and Their Relation to Domain Topology Probed by Single-Molecule FRET
Source: Biomolecules. 2023 Aug 22;13(9):1280. doi: 10.3390/biom13091280 (PMC10526189; doi:10.3390/biom13091280)
Supplement: Supplementary file 1 [file biomolecules-13-01280-s001.zip › biomolecules-2502353-supplementary.pdf]

Supplementary Materials

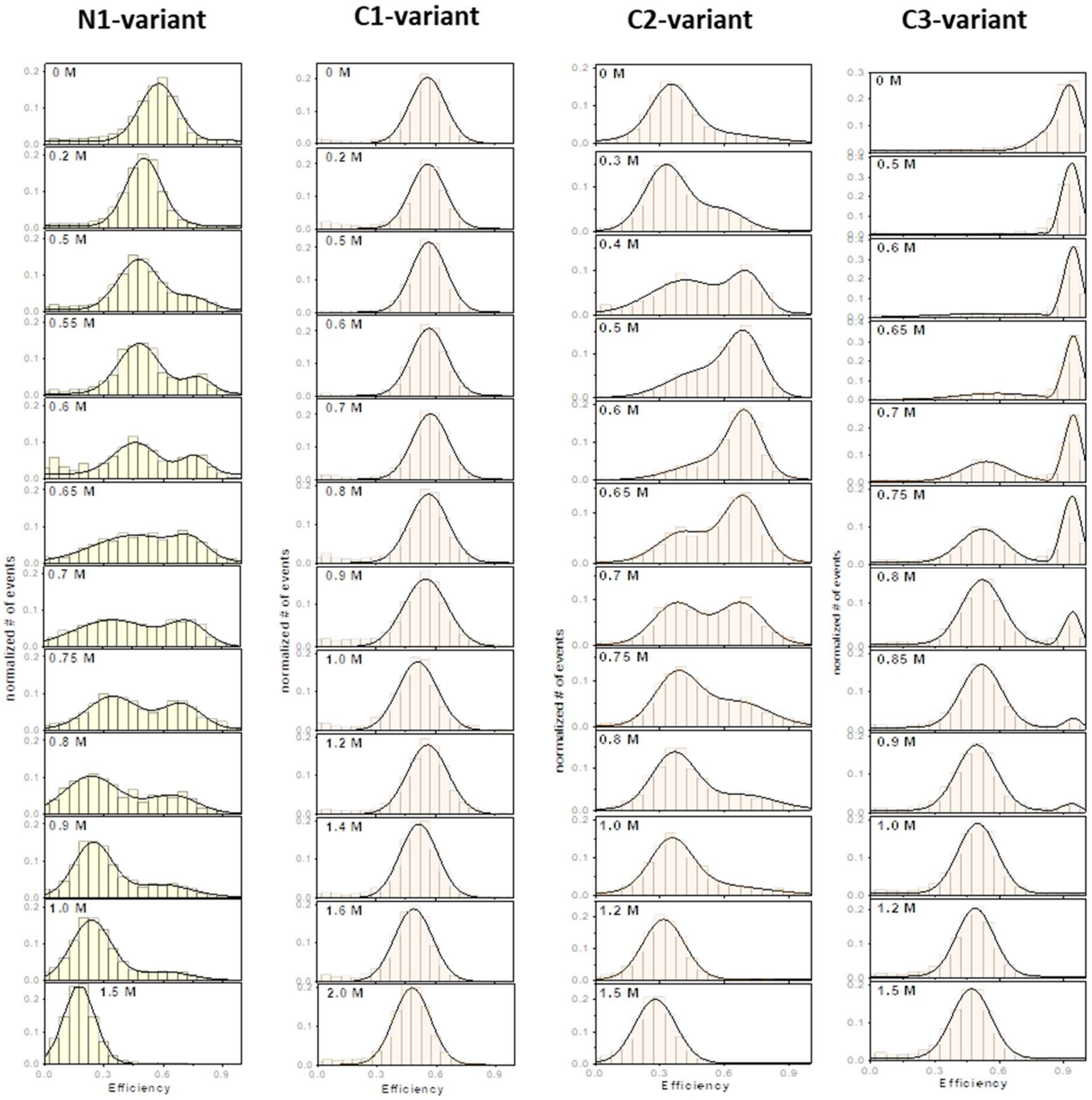

Figure S1: Single-molecule FRET histograms, mainly taken from data published in ref. [4]

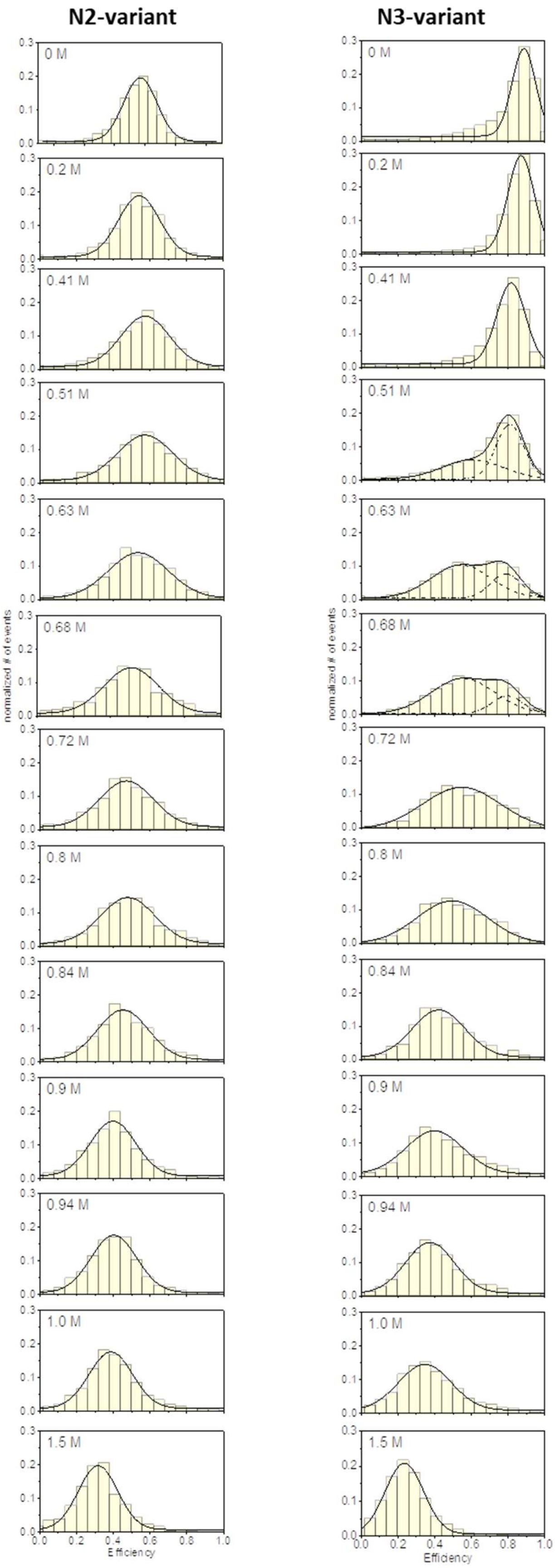

Figure S2: Single-molecule FRET histograms obtained from measurements performed in this study.

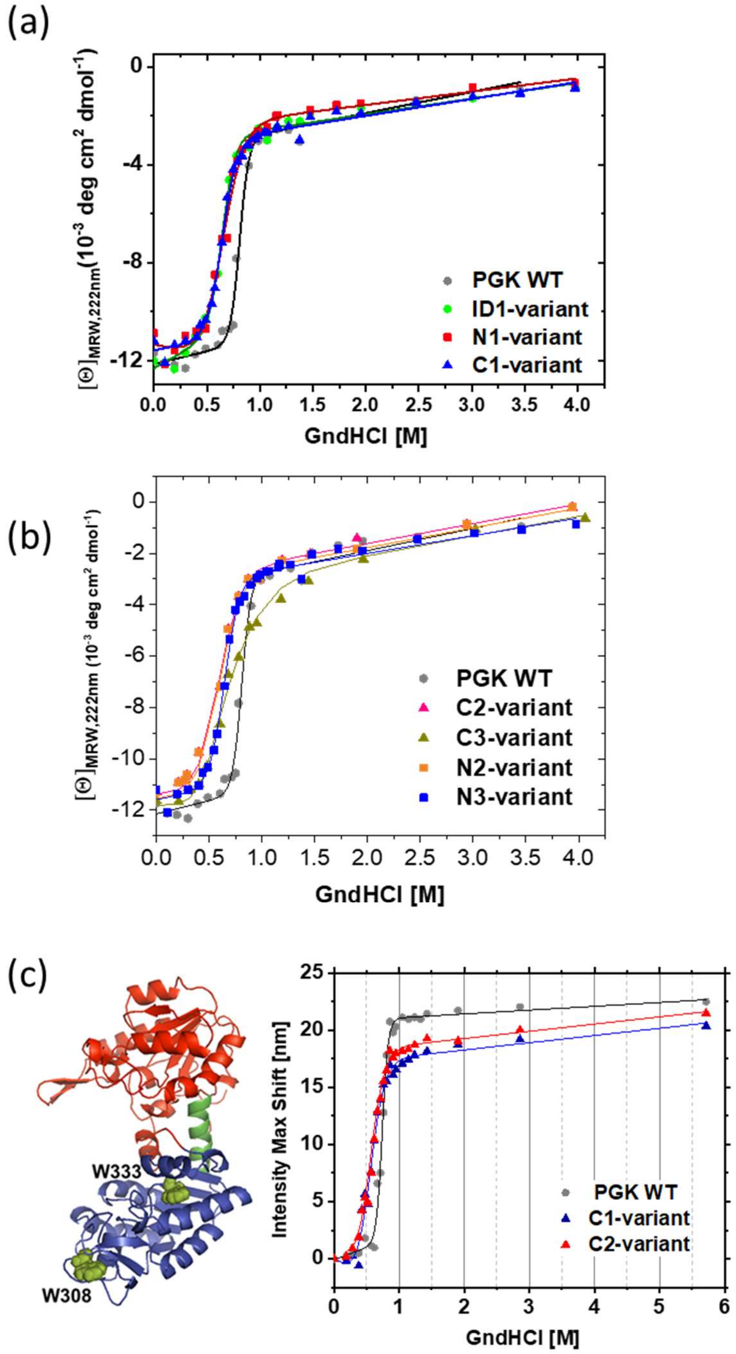

Figure S3: (a,b) Ellipticity peak values, obtained from CD-spectroscopy measurements at 222 nm, are plotted as a function of GndHCl concentrations for the yPGK wild-type (WT) and for all variants (including a variant which probes the a inter-domain distance ID1: Q135C-S290C, for details see ref. [4]. The measured data points (symbols) where fitted with a thermodynamic two-state model (solid line). (c) Intrinsic tryptophan fluorescence was employed to follow the tertiary structural integrity of the C-terminal domain of yPGK upon denaturant induced unfolding (there are only two tryptophan residues in the C-terminal domain). Also these data points were fitted with the same model as in a,b (for methodical details see ref. [4]. Data partly taken from ref. [4].
